# Peritraumatic dissociation predicts posttraumatic stress disorder symptoms via dysfunctional trauma-related memory among war-affected children

**DOI:** 10.1080/20008198.2017.1375828

**Published:** 2017-10-10

**Authors:** Kirsi Peltonen, Samuli Kangaslampi, Jenni Saranpää, Samir Qouta, Raija-Leena Punamäki

**Affiliations:** ^a^ University of Tampere, Finland/Faculty of Social Sciences/Psychology, Tampere, Finland; ^b^ Lapua Developmental and Family Counselling, Lapua, Finland; ^c^ Islamic University of Gaza, Department of Education and Psychology, Gaza

**Keywords:** War trauma, children, peritraumatic dissociation, trauma-related memory, posttraumatic stress disorder, trauma de guerra, niños, disociación peritraumática, recuerdo relacionado con el trauma, trastorno de estrés postraumático, 战争创伤, 儿童, 围创伤期分离, 创伤相关记忆, 创伤后应激障碍, • Children’s self-reported dissociation (PD) during their exposure to recent war trauma predicted posttraumatic stress (PTSD) symptoms nine months post-war., • The association between PD and PTSD symptoms was partially mediated by dysfunctional memory processing of trauma., • The war impacts may be symptom-specific, as high exposure correlated with intrusive and dysphoria symptoms, but not with avoidance or hyperarousal., • The nature of traumatic events may matter more than the sole numbers, as, for instance, witnessing horrifying scenes with multiple sensory stimuli may form especially severe risk for intrusive symptoms.

## Abstract

**Background**: Among adults there is strong evidence about peritraumatic dissociation (PD) predicting posttraumatic stress disorder (PTSD), yet evidence among children is very limited. It has been suggested that disturbances in memory functioning might explain the association between PD and PTSD, but this has not yet been empirically tested.

**Objective**: We aimed to test the hypotheses that greater PD would be associated with more posttraumatic stress disorder (PTSD) symptoms, and that some of this association would be mediated by disorganized and non-verbal memories about the traumatic event.

**Method**: The sample included 197 Palestinian children (10–12-years) living in the Gaza Strip, participating in the aftermath of the 2008/9 war. Self-report questionnaires were used to measure PD (Peritraumatic Dissociative Experiences Questionnaire) three months post-war, as well as trauma-related memory (Trauma Memory Quality Questionnaire) and PTSD symptoms (Children’s Revised Impact of Event Scale) six months later. Exposure to war trauma was assessed by a checklist. Structural equation modelling was used to examine direct and indirect paths from PD to posttraumatic PTSS, controlling for number of traumatic war events.

**Results**: Structural equation modelling results showed that greater self-reported PD predicted higher levels of PTSS nine months post-war, and that a significant part, but not all, of this relationship was mediated via the quality of trauma-related memories.

**Conclusions**: This study provided empirical evidence that, among war-affected children, greater PD during traumatic events is linked with higher levels of PTSD symptoms several months later, even when accounting for their personal exposure to war trauma. Further, the study supported the idea that the detrimental effects of dissociation during a traumatic event may be due to dysfunctional memories characterized by disorganization and lack of access to verbal and coherence. Further tests of these hypotheses with larger samples and more points of measurement are called for.

## Abstract (es)

**Planteamiento:** Entre los adultos existe mucha evidencia sobre la disociación peritraumática (DP) que predice el trastorno de estrés postraumático (TEPT); sin embargo, la evidencia en niños es muy limitada. Se ha sugerido que las alteraciones en el funcionamiento de la memoria podrían explicar la asociación entre la DP y el TEPT, pero esto aún no ha sido probado empíricamente.

**Objetivo:** Se intentó probar la hipótesis de que una mayor DP estaría asociada con síntomas de trastorno de estrés postraumático (TEPT), y que parte de esta asociación estaría mediada por recuerdos desorganizados y no verbales sobre el evento traumático.

**Método:** La muestra incluyó a 197 niños palestinos (10–12 años) que vivían en la Franja de Gaza, que vivieron las secuelas de la guerra de 2008/9. Se utilizaron cuestionarios de auto-informe para medir la DP (Peritraumatic Dissociative Experiences Questionnaire) tres meses después de la guerra, así como la memoria relacionada con traumas (Trauma Memory Quality Questionnaire) y los síntomas de TEPT (Children’s Revised Impact of Event Scale) seis meses después. La exposición al trauma de guerra se evaluó mediante una lista de verificación. Se utilizó el modelado de ecuaciones estructurales para examinar las trayectorias directas e indirectas de la DP al TEPT, controlando el número de eventos de guerra traumáticos.

**Resultados:** Los resultados del modelado de la ecuación estructural mostraron que los auto-informes de mayor DP predecían niveles más altos de TEPT nueve meses después de la guerra, y que una parte significativa, pero no toda, de esta relación fue mediada por calidad de los recuerdos relacionados con el trauma.

**Conclusiones:** Este estudio proporcionó evidencia empírica de que, entre los niños afectados por la guerra, una mayor DP durante los eventos traumáticos está relacionada con niveles más altos de síntomas de TEPT varios meses después, incluso cuando se explica su exposición personal a un trauma de guerra. Además, el estudio respalda la idea de que los efectos perjudiciales de la disociación durante un evento traumático pueden ser debidos a los recuerdos disfuncionales caracterizados por desorganización y falta de acceso a lo verbal y a la coherencia. Se requieren más pruebas de estas hipótesis con muestras más grandes y más puntos de medición.

## Abstract (zh)

**背景：**在成人中有强证据支持围创伤期分离（PD）预测创伤后应激障碍（PTSD），但是在儿童中的证据有限。记忆功能受损或许可以解释PD和PTSD的关联，但还未被实证验证过。

**目标：**我们想要验证更高PD和更多PTSD症状关联的假设，其中有些关联路径可能被对创伤事件的杂乱的和非语言性记忆中介。

**方法：**样本包括了197名住在加沙地带的巴勒斯坦儿童（10–12岁），在2008年9月的战争后参加研究。用围创伤期分离体验问卷（Peritraumatic Dissociative Experiences Questionnaire）测量战后三个月的PD，创伤记忆质量问卷Trauma Memory Quality Questionnaire）测量创伤相关记忆，修订后儿童版事件影响量表（Children’s Revised Impact of Event Scale）测量6个月后的PTSD。并使用一个清单量表评测对战争创伤的暴露。使用结构方程模型来考察控制战争创伤事件后PD到PTSS直接和间接的路径。

**结果：**结构方程模型的结果显示更多地自我报告PD预测9个月后更高水平的PTSS，这种关联中的大部分（并非全部）被创伤相关记忆的质量中介了。

**结论：**本研究提供了实证证据支持在受战争影响的儿童中，经历创伤事件时更多PD和几个月之后更高水平的PTSD症状有关联，在控制了个体的战争创伤暴露程度之后也如此。进一步地，本研究支持了分离症状在创伤事件中的有害影响可能是因为异常记忆通常表现出的混乱和难以用语言表达和缺乏连贯性。未来需要在更大样本中使用更多测量点对这些假设进行进一步验证。

## 1. Introduction

Cumulative traumatic experiences increase the risk for psychiatric disorders and symptoms such as posttraumatic stress disorder (PTSD), generalized anxiety, traumatic grief, and depression among children living in war areas (Betancourt et al., 2012; Catani et al., 2009). The prevalence of PTSD, however, varies considerably among war-affected children. For instance, among Palestinian children exposed to major wars in the Gaza Strip, the prevalence of PTSD varied between 22% and 34% (Altawil, Nel, Asker, Samara, & Harrold, 2008; Thabet & Vostanis, 2015). Researchers are interested in explanations for such variation and are especially seeking early risk factors that predict the diagnosis of PTSD among children (Dalgleish et al., 2008). Research suggests pre-, peri-, and post-trauma responses to be decisive for the development of PTSD (Olff et al., 2015), with acute stress disorder (ASD) and peritraumatic dissociation (PD) playing an important role (Kassam-Adams et al., 2012). Among adults there is evidence about PD predicting PTSD (for a meta-analysis, see Lensvelt-Mulders et al., 2008; Ozer, Best, Lipsey, & Weiss, 2003), especially in the short run (Thomas, Saumier, & Brunet, 2012; van der Velden & Wittman, 2008). Yet, among children and adolescents evidence is very limited and findings are inconclusive, as some research among young vehicle-accident survivors has confirmed that PD forms a severe risk for PTSD (Bui et al., 2010), while others showed minor importance (Dalgleish et al., 2008). We could not find research on PD among children in war conditions. The present study analyses the association of PD and PTSD symptoms among Palestinian children in the aftermath of a major war, the 2008/9 War on Gaza.

Excessive trauma can overwhelm children’s capacity for integrating sensations, emotions, and thinking into coherent memories, resulting in a high risk for trauma-related disorders, especially PTSD (Dalgleish et al., 2008; Ehlers & Clark, 2000; Meiser-Stedman, Dalgleish et al., 2007). Dissociative responses are an expression of the ‘shut-down’ of normal information processing during the traumatic event. PD involves a distorted sense of time and place and leads to a sense of unreality, appearing as depersonalization with a lack of subjective emotions, out-of-body experiences, and altered pain perceptions (Marmar, Weiss, & Metzler, 1998). Disruptions of memory are common and distortions of time, either as decelerated or accelerated, may be experienced (American Psychiatric Association, 2013; Schauer & Elbert, 2015).

Biological and psychosocial mechanisms have been suggested to explain the association between PD and PTSD (Apfel et al., 2011; Schauer & Elbert, 2015), with disturbances in memory functioning providing one promising approach (Bedard-Gilligan & Zoellner, 2012; Spiegel, Koopmen, Cardena, & Classen, 1996). The corticolimbic model of dissociation suggests that in a state of high anxiety, the prefrontal cortex inhibits emotional processing in limbic structures such as the amygdala. Subsequently, sympathetic output is dampened and emotional experiencing is reduced (Lanius, Bluhm, Lanius, & Pain, 2006). Subsequently, trauma-related memories are fragmented and lack chronological order, consistency, and narrative quality. Victims are later able to retrieve predominantly sensory-based memories of images, voices, odours, or feelings that are not verbally accessible (Engelhard, van den Hout, Kindt, Arntz, & Schouten, 2003; Meiser-Stedman, Smith, Yule, & Dalgleish, 2007). They face difficulties consciously recalling a coherent, chronological memory about the event but instead experience impulsive sensations that may uncontrollably invade the mind (Brewin, Dalgleish, & Joseph, 1996; Ehlers & Clark, 2000). Encounters with trauma reminders may trigger the same dissociative responses experienced during the trauma, which in turn maintain memory dysfunction. Theorists suggest that PTSD develops, at least partly, as a consequence of the traumatized person’s inability to process the event at a symbolic and verbal level (Brewin et al., 1996; Ehlers & Clark, 2000). However, the pathway from PD to PTSD via dysfunctional trauma-related memories has not been tested, to our knowledge, among children.

### 1.1. Research questions

We examined whether peritraumatic dissociation (PD) associates with dysfunctional trauma-related memory and PTSD symptoms and whether the trauma-related memory mediates the association between PD and PTSD symptoms. Palestinian children were followed from three months after a major war (T1) until nine months post-war (T3). We hypothesized that: (1) Child-reported peritraumatic dissociation at T1 will predict their reported PTSD symptoms at T3; and (2) dysfunctional trauma-related memories mediate the association between PD and PTSD symptoms.

## 2. Method

### 2.1. Participants and procedure

The participants were 197 Palestinian schoolchildren living in the Gaza Strip (10–12 years, M = 11.35, SD = .57; 49.4% girls), and derived from the control group (n = 240). They formed the control group in a randomized controlled trial of an intervention after the 2008/9 War on Gaza (Kangaslampi, Punamäki, Qouta, Diab, & Peltonen, 2016). Participants completed the measurements three months after the war at baseline, two months after the intervention, and at a six-month follow-up. The subsample of 197 consists of children who have complete data from assessments at baseline (T1) and follow-up (T3). The ethical boards at the Palestinian Ministry of Education and the Gaza Community Mental Health Programme (GCMHP) reviewed and accepted the study protocol and measurements. Permissions for the study were received from school authorities. Information sheets were prepared for the children and their parents explaining the purpose of the study, and only verbal consent to participate in the study was required. Research assistants conducted the fieldwork in school classes and children received detailed verbal and written instructions before they completed questionnaires. GCMHP provided consultation to parents and teachers who had high levels of worry concerning the children in the aftermath of the war.

All questionnaires and instructions were administered in Arabic. Measures of War trauma, PD, and PTSD symptoms were available in Arabic. The measure of trauma-related memory was translated and back-translated from and to English by the bilingual researchers. All scales were pilot-tested.

### 2.2. Measures

#### 2.2.1. War trauma at T1

A checklist of 29 traumatic events was constructed for this study. It covers typical events during the War on Gaza 2008/9 and the military siege related to witnessing acts of violence, losses, injury and destruction, and being personally the target of violence. Children reported at T1 whether they had experienced each traumatic event (1 = *yes*; 0 = *no*). A sum variable was constructed by counting the affirmative answers to 14 questions about events fulfilling the DSM-IV definition of trauma.

#### 2.2.2. Peritraumatic dissociation at T1

The 10-item Peritraumatic Dissociative Experiences Questionnaire (PDEQ; Marshall, Orlando, Jaycox, Foy, & Belzberg, 2002) describes experiences of depersonalization, thought confusion, and lack of emotion. It has been validated in different cultural contexts, and good psychometric properties were found e.g. among war-traumatized Ugandan children (Klasen et al., 2010). Children were asked to recall their experiences during the last war, and report whether they had each type of dissociative responses (1 = *yes*; 0 = *no*). A sum variable was constructed. Cronbach’s α = .71.

#### 2.2.3. Trauma-related memory at T3

The 11-item Trauma Memory Quality Questionnaire (TMQQ; Meiser-Stedman, Smith et al., 2007) was applied to assess children’s memory quality. This self-report questionnaire inquires about the ways survivors remember the traumatic event. For example: **‘**My memories of the frightening event are mostly pictures or images’. Children evaluated on a 4-point scale how well the statements fit them (1 = *not at all*; 4 = *completely*). A sum variable was constructed, and the value of α was .73.

#### 2.2.4. Posttraumatic stress disorder (PTSD) symptoms at T3

PTSD symptoms were measured by the 13-item Children’s Revised Impact of Events Scale (CRIES; Smith, Perrin, Dyregrov, & Yule, 2003). On this self-report questionnaire, based on DSM-IV criteria, children evaluated on a 4-point scale how often they had experienced a particular symptom over the last two weeks (0 = *not at all*, 1 = *rarely*, 3 = *sometimes*, 5 = *often*). CRIES has shown adequate reliability and validity in Palestinian samples (Kolltveit et al., 2012). Cronbach’s α was .63

### 2.3. Statistical analysis

The current study is a secondary analysis of the sample, testing the hypothesis of dysfunctional trauma-related memory mediating the association between PD and PTSD symptoms. The 197 children for whom follow-up measurements were available were included in this study. There were no missing individual data points.

Structural equation modelling with a mix of latent and manifest variables was used to estimate direct and mediated effects. Posttraumatic stress symptoms were modelled as a latent variable. Based on earlier analyses with this data (Palosaari, Punamäki, Diab, & Qouta, 2013), a four-factor structure similar to that identified among combat veterans (Simms, Watson, & Doebbeling, 2002) was selected for this purpose. Parcel indicators were constructed corresponding to intrusion (average of four items on the CRIES), avoidance (four items), dysphoria/negative affectivity (three items) and hyperarousal (two items) symptoms. Peritraumatic dissociation, number of traumatic war events, and trauma-related memory were treated as manifest sum variables.

The fit of a measurement model, where all variables were allowed to covary freely, was first evaluated. A structural model with constraints representing the study hypotheses was then applied to create the final model. In this structural model, posttraumatic stress symptoms were regressed on peritraumatic dissociation, trauma-related memory, and war trauma, while trauma-related memory quality was regressed on peritraumatic dissociation. The fit of the final model and its estimated parameters were then evaluated.

All analyses were carried out using R (R Core Team, 2016) and the lavaan package (Rosseel, 2012). For structural equation modelling, full information maximum likelihood estimation with bootstrapped standard errors from 5000 resamples was used. Asymmetric confidence intervals based on bias-corrected bootstrap estimates were constructed for total, indirect, and direct effects to ensure accurate assessment of their significance and take into account the non-normal distribution of the indirect effect (MacKinnon, Lockwood, & Williams, 2004).

## 3. Results

### 3.1. Descriptive results

Out of an original sample of 240 participants 49% were girls. A majority lived in urban areas (86%), 12% in refugee camps, and 3% in villages. A quarter of fathers (25%) and 8% of mothers had university-level education, while 24% of fathers and 42% of mothers had only finished secondary level. Mothers were on average 37.6 and fathers 42.4 years old. There was a high rate of unemployed fathers (49%), which corresponds with general Palestinian statistics in the Gaza Strip during the international siege and economic blockade (UN OCHA, 2009). Most of the mothers (98%) worked at home. The number of traumatic events experienced by the participating children varied between 1–21, the mean and the median being eight events.

Out of the 240 children assessed at T1, T3 measurements were available for 197. There were no refusals to participate at T3 – instead, dropouts were children who changed schools, dropped out of school altogether or were not present at school during measurements at T3 for other reasons. There were no significant differences between dropouts and those remaining in age, number of traumatic experiences or PTSS at T1. However, significantly more boys than girls dropped out before T3 (25.2% boys vs. 14.9% girls, *χ^2^* (1) = 8.54, p = .003), and dropouts reported more peritraumatic dissociation (*M* = 7.19 vs. *M* = 6.17, 95% CI_diff_ [.07, 1.96], *t* (238) = 2.23, *p* = .03). Controlling for gender, dropout did not significantly predict level of peritraumatic dissociation, suggesting that the difference was mostly explained by higher peritraumatic dissociation among boys overall.

### 3.2. Direct and mediated effects

Table 1 presents the bivariate correlations between variables included in the structural equation model. The measurement model fit the data well (*χ^2^* (11) = 12.53, *p* = .325, RMSEA = .02, 90% CI [.00, .08], CFI = 0.99, TLI = 0.98, SRMR = .04). The final structural equation model also had a good fit (*χ^2^* (12) = 12.84, *p* = .381, RMSEA = .02, 90% CI [.00, .08], CFI = 0.99, TLI = 0.99, SRMR = .04).

**Table 1.** Zero-order bivariate correlations between peritraumatic dissociation, trauma memory quality, traumatic events, and posttraumatic stress symptoms.

	Measure	1	2	3	4	5	6
1	Peritraumatic dissociation						
2	Trauma memory quality	.33***					
3	Traumatic events	.22**	.11				
4	Intrusions	.29***	.41***	.16*			
5	Avoidance	.07	.23**	−.03	.20**		
6	Dysphoria	.20**	.25**	.17*	.25**	.02	
7	Hyperarousal	.14*	.27***	.10	.22**	.07	.25***

*N *= 197. * *p* < .05; ** *p* < .01; *** *p* < .001

**Figure 1.**
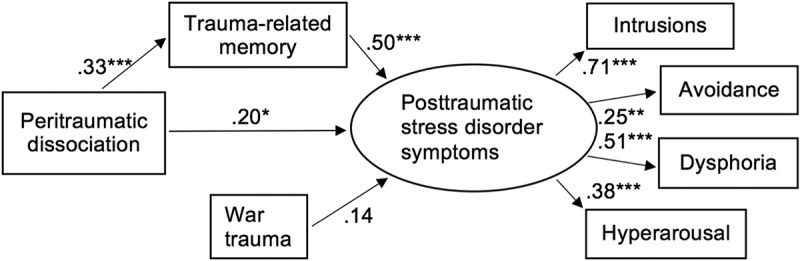
Structural equation model of trauma-related memory mediating the effect of peritraumatic dissociation on posttraumatic stress disorder symptoms. Fully standardized maximum likelihood estimates of path coefficients presented. Residual variances omitted for clarity. *Note*: *p < .05; **p < .01; ***p < .001

The final model with estimated parameters is shown in Figure 1. The final model explained 40.0% of the variance in the latent posttraumatic stress symptoms variable (R^2^ = .399, *p* < .0001). As hypothesized, PD at three months post-war significantly predicted PTSS six months later (unstandardized total effect = .094, 95% CI [.046, .15]; fully standardized effect = .36, *SE* = .09, *p* < .0001). In line with our second hypothesis, peritraumatic dissociation had an indirect effect on posttraumatic symptoms via effects on trauma memory quality (unstandardized indirect effect = 0.043, 95% CI [.023, .073]; fully standardized effect = .17, *SE* = .05, *p* = .001). However, even taking this indirect effect into account, the direct path from PD to PTSS was also still significant (unstandardized effect = .051, 95% CI [.004, .10]; fully standardized effect = .20, *SE *= .09, *p *= .03), indicating partial, but not total, mediation.

## 4. Discussion

Dissociation during exposure to traumatic events is considered a severe risk for the survivor’s mental health, and evidence of this is substantial among adults (e.g. Ozer et al., 2003). Along these lines, our results showed that children’s self-reported dissociation (PD) during their exposure to recent war trauma predicted posttraumatic stress (PTSD) symptoms nine months post-war. Our findings further confirmed that the association between PD and PTSD symptoms was partially mediated by dysfunctional memory processing of trauma. This is in accordance with theories suggesting that risks and maintenance of PTSD are due to deficient encoding of traumatic memories and later dysfunctional recalling and processing of events, as well as failure in symbolization and integration of traumatic scenes (Brewin, Dalgleish, & Joseph, 1996; Ehlers & Clark, 2000).

The importance of PD in predicting PTSD among these Palestinian children concurs with adult survivors of war and peace-time traumas (Lensvelt-Mulders et al., 2008; Thomas et al., 2012) but differs from some findings among children exposed to single peace-time trauma (Dalgleish et al., 2008). The importance of PD as a severe risk for PTSD symptoms among war-affected Palestinian children may lie in the chronicity of life threat and adversities. It is possible that participating children faced multiple reminders of threats and horrors, which activated their war-related vulnerabilities. Due to the international boycott and Israeli military siege their families continued to live in highly insecure and deprived conditions interfering with optimal recovery, family life, and trauma processing.

As hypothesized, dysfunctional trauma-related memories mediated the association between PD on PTSD symptoms. These memories were typically uncontrollable and fragmented, and often brought back horrific scenes with possible strong sensory and procedural cues, such as odours or urgency to flee. We could not detect previous mediation analyses with trauma-related memories explaining the importance of PD on PTSD, but there is evidence of avoidant coping style and thought suppression of the traumatic event as mediators (Engelhard et al., 2003; Pacella et al., 2011), as well as loss of control and feelings of helplessness and life-threat (Gershuny, Cloitre, & Otto, 2003). These results indicate, similar to ours, that peritraumatic dissociations negatively alter survivors’ attempts to process their traumatic experiences, which in turn form a mental health. Further studies should simultaneously test multiple mediating paths between children’s dissociation during trauma and various mental health and developmental problems.

In our model of trauma-related memories mediating between PD and PTSD symptoms, children’s personal exposure to war trauma was not associated with the latent construct of PTSD symptoms nine month afterwards. Yet, correlation analysis revealed that the war impacts may be symptom-specific, as high exposure correlated with intrusive and dysphoria symptoms, but not with avoidance or hyperarousal. Analysis also showed that avoidance symptoms did not correlate with other PTSD symptoms, which may hint at their psychological functionality in chronic insecure and adverse conditions. Earlier findings among Palestinian children similarly showed that the accumulation of traumatic war events was not a significant predictor of PTSD symptoms (Peltonen, Qouta, El Sarraj, & Punamäki, 2010). We suggest that the nature of traumatic events matters more than the sole numbers, as, for instance, witnessing horrifying scenes with multiple sensory stimuli may form especially severe risk for intrusive symptoms. Clinically, it would be pivotal to know how children remember the traumatic situation, what meanings and associations they incorporate to it, and how they have attempted to cope with and process the trauma.

Knowledge about the cognitive, emotional, and psychophysiological processes underlying the link between trauma and PTSD and other symptoms is pivotal when tailoring therapy and treatment for war-affected children. Cognitive behavioural therapy (CBT) approaches aim to integrate shattered psychic processes, including trauma memories, into integrated and controllable life histories (Peltonen & Punamäki, 2010). However, treatment for dissociation is not a routine element in these treatments (Schauer & Elbert, 2015). Our results on both direct and partially mediated impacts of PD on posttraumatic symptoms suggest that children might need specific help in dealing with and processing dissociative states that may be constantly re-evoked in post-war conditions. Severe PD interferes with optimal recovery from trauma, which requires multimodal and symbolic processing of traumatic experiences.

This research deserves criticism for the self-reported nature of the measures and the partly cross-sectional setting. A review concerning adult patients concluded that the association between dissociation and memory dysfunction was significant when based on self-ratings, but less prominent when rater-coded or computer-generated measures were used (Bedard-Gilligan & Zoellner, 2012). The results should thus be replicated in a setting with clinical interviews, observed dissociation data, and better measures of memory fragmentation and bias. Further, the fact that trauma-related memory quality and PTSD symptoms were assessed at the same time is an important limitation for this mediation analysis. Although a measure of PTSD symptoms used in this study (CRIES) has shown adequate reliability and validity in Palestinian samples (Kolltveit et al., 2012), it showed relatively modest reliability in this trial.
